# Frame Synchronization of High-Speed Vision Sensors with Respect to Temporally Encoded Illumination in Highly Dynamic Environments

**DOI:** 10.3390/s130404102

**Published:** 2013-03-26

**Authors:** Lei Hou, Shingo Kagami, Koichi Hashimoto

**Affiliations:** 1 Department of Automation, Shanghai Jiao Tong University, and Key Laboratory of System Control and Information Processing, Ministry of Education of China, Shanghai 200240, China; 2 Graduate School of Information Sciences, Tohoku University, 6-6-01 Aramaki Aza Aoba, Aoba-ku, Sendai 980-8579, Japan; E-Mails: swk@ic.is.tohoku.ac.jp (S.K.); koichi@ic.is.tohoku.ac.jp (K.H.)

**Keywords:** robot vision, vision chip, camera synchronization, visual feedback control, phase-locked loop, signal normalization, quadrature detection, intelligent coding, Manchester encoding

## Abstract

The authors propose a Manchester Encoding inspired illumination modulation strategy to properly index the temporally-aligned vision frames, which are successfully synchronized by the LED reference signal. Based on signal normalization, Manchester Encoded reference signals carry temporal information owing to serial communication and thus can timestamp the output vision frame. Both simulated and experimental results show satisfactory robustness to various disturbances, such as dynamic targets, fluctuant optical intensity, and unfixed cameras, *etc*. The 1,000 Hz vision sensor is locked to 500 Hz temporally modulated LED illumination with only 24 *μ*s jitters. This result is believed to be applicable to low-cost wireless vision sensor network.

## 1. Introduction

The emphasis of vision sensor technology becomes more and more evident in various visual measurements, such as automotive, human machine interface, surveillance and security, and industry control. For example, if we introduce a vision sensor for high-speed visual information [1] and proposed an appropriate control algorithm for the vision sensor utilizing some unique features, real-time visual measurement [2] and wearable biometrics devices will be achieved. Ideally, synchronization can be achieved without any external triggers or references in the computer vision field. Multiple groups of images bring much more valuable additional information, such as the depth parameter, to perform accurate measurements in the real world, without the limitation of one-view measurement techniques [3].

Firstly, there are a group of studies in which geometric correspondences such as points are used for synchronization [4–11]. Although these methods can carry out geometric calibration and synchronization simultaneously, a sufficient number of correspondences across images are necessary. This is not appropriate depending on applications. Also, estimating simultaneously geometric parameters and time delays, which are inherently independent of each other, might sacrifice accuracy to some degree.

Therefore, it is more desirable to synchronize without using image correspondences. Yan and Pollefeys proposed a method for video synchronization [12] that uses the space-time interest points defined by Laptev and Lindeberg [13]. This method also fails to synchronize images in the case of foreground objects [14]. When the feature points are not available or reliable, some alternative algorithms that use the object outline or silhouette as the reliable image feature exploit into the epipolar tangents [15], *i.e.*, points on the silhouette contours in which the tangent to the silhouette is an epipolar line [16]. A rich literature exists on exploiting epipolar tangents, both for orthographic cameras [15,17] and perspective cameras [18]. There are also factorization-based methods to recover 3D models from multiple perspective views with uncalibrated cameras, performing a projective reconstruction using a bilinear factorization algorithm and then converting the projective solution to a Euclidean one by enforcing metric constrains, but they are based on static scenes and moving objects [11, 19– 21]. Some contributions are devoted to comparing the probability of distributions [22,23]. In [14], a method for the time synchronization of a multiple-camera system is proposed without using an external clock signal. The basic idea is to use co-occurrence of appearance changes of objects in motion that are observed on different views. Specifically, the spatial integral over the image plane of temporal derivatives of brightness is used as a temporal feature of a video sequence. Although a great amount of efforts have been devoted to the image-based synchronization technique, they are not universal and may not be applicable in the real world applications due to the innate limitations, such as prerequisite LED auxiliary, arbitrarily tilting or stationary cameras, specific texture of background, or restrictive motion of objects.

Actually, camera synchronization with external clocks or triggers is still needed in the practical viewpoint. Generally, there are three categories of state-of-the-art techniques. The first is to use dedicated wires to transfer the reference signal. Many of the industrial vision sensors are equipped with dedicated electrical inputs/outputs to synchronize trigger signals, in which one of the vision sensors—or a dedicated signal emitter device—acts as a master, and the others are operated in synchronization with the trigger signal emitted from the master. A major problem in this classical and widely-used means is that deployment of synchronization wires is cumbersome in some situations—short wires may impose constraints on spatial configuration of vision sensors; long wires may cause unstable synchronization. The second solution is to use wired standard bus such as IEEE1394 and Ethernet. Instead of dedicated synchronization wires, some systems allow synchronization through standard electronic buses used for image transfer such as IEEE 1394 [24] and Ethernet [25,26]. These systems bring higher flexibility, but they still require wired connections and are unsuitable for wireless vision sensor networks. The third type is to employ wireless communication protocols for synchronization in sensor network field. The principal difficulty in time synchronization of wireless network systems lies in nondeterminism in wireless media access time [27]. Due to this nondeterminism, it is difficult to make certain when a synchronization packet started to propagate from the sender. RBS [28] introduced a receiver-receiver synchronization scheme to remove the effect of the sender nondeterminism, but requires many message exchanges between receivers to achieve high precision. TPSN [29] and FTSP [30] suppress this nondeterminism by time stamping at the media access control (MAC) layer, but they inherently require special MAC implementations. It is also possible to equip a dedicated receiver of radio or optical reference synchronization signal, but at the cost of additional equipments.

This paper proposes to use temporally encoded illumination instead of regularly modulated illumination for camera synchronization that can be used even for low-cost wireless vision sensor networks.

**Figure 1.**
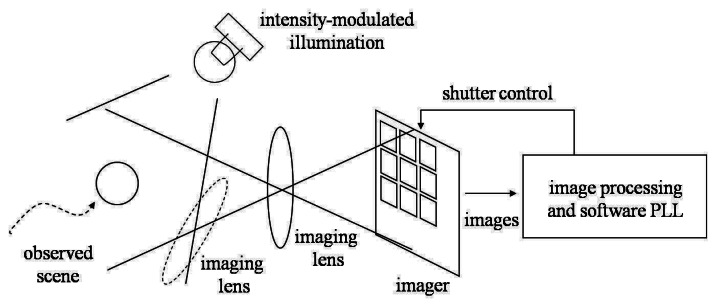
Conceptual diagram of the illumination-based camera synchronization system.

Figure 1 illustrates the conceptual diagram of the proposed synchronized camera system. Incident light to cameras serves as the reference signal. Internal functions of cameras, such as the analog photo integration process in the imager and the digital computation executed outside the imager, constitute a PLL to synchronize the output signal, which is the vision frame timing. In this way the camera frame timing is locked to the reference illumination.

This method can time stamp the synchronized camera frames with the serially encoded illumination other than just aligning the timing of the frames. Previously, the illumination-based synchronization technique with regularly intensity modulated illumination does not carry any time information yet. Though the facility of frame index is not always indispensable, it will certainly expand the application domain. There are many industrial cameras equipped with wired synchronization trigger inputs/outputs, which send/receive only triggers for shuttering timing but without information on frame correspondence, nevertheless they are still useful in various applications. Although there are many state-of-the-art researches into temporal index techniques on wireless communication, such as [31,32], we expect to develop the most natural and unaffected temporal index scheme to identify the time information taken by vision sensors. Fortunately, this issue can be addressed with the help of serial communication.

## 2. Synchronization Algorithm

**Figure 2.**
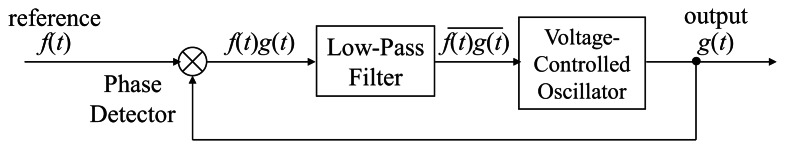
Block diagram of a PLL with PI controller.

Figure 2 shows a standard PLL feedback system in which the output signal *g*(*t*) is synchronized to the reference *f*(*t*) in phase as well as in frequency, as introduced in [33]. Exploiting that *g*(*t*) is a constant during a frame period, the time correlation $\bar{f(t)g(t)}$ can be computed as

(1) $$\bar{f(t)g(t)}\propto \sum_{i}{\left(-1\right)}^{i-1}F[i]$$

where *i* is the frame number index and *F*(*i*) is the sum of the pixel values obtained within the frame *i*.

However, although this synchronization technique is mature, it is still necessary to add the frame index technique. Without clear index information of the images taken by each vision sensor, it is difficult to recognize the right sequence of all the images. We can index the vision frame by modulating the reference signal in the Manchester Encoding strategy. For the sake of shutter time synchronization, actually for time correlation purpose, illumination modulation strategy can be derived from many intelligent coding sequences, such as Manchester Encoding sequence, Pseudo-Random number sequence, Barker sequence, *etc*. The transition property of Manchester Encoding makes it the most straightforward strategy for the wireless illumination-based synchronization of vision sensors, because its transition property ensures that there always exist appropriately enough bright reference unit durations within every reference period to serve as the light source, as well as to successfully synchronize the shutter time. Temporal modulation can also be based on alternative coding strategies, such as the Pseudo-Random sequence and the Barker Sequence. At the current stage, Manchester Encoding strategy is effective to test the feasibility of the time index function of vision sensors. Pseudo-Random number generator and the Barker Sequence stand for more exquisite and automatic techniques. More robust and complicated functions may be realized later owing to the excellent properties and higher performances of these coding strategies.

The index denoted by a group of *On* or *Off* images corresponds to the sequence of vision frames respectively. A straightforward strategy is to define *g*(*t*) so that it takes values 1, 1, −1, −1, 1, 1, −1, and −1, during the frames whose frame number mod 8 is equal to 0, 1, 2, 3, 4, 5, 6, and 7 respectively. Using the time correlation value $q=\bar{f(t)g(t)}$, we can estimate that the reference amplitude is proportional to *q*, by which the feedback control is possible.

Unfortunately, this is not the best choice when we account for the existence of a non-photo-integration period within a frame time. In the locked state with the above definition, a rising or falling edges of the reference signal comes just between a frame and a frame, which is in most cases within a non-integration period. Since no measurement is done in a non-integration period, it is impossible to distinguish the locked state from any situations in its neighbor—specifically the situations where all of the rising and falling edges are within non-integration periods. In other words, the time correlation is not sensitive to the small phase error around *ø*=*π*/2. This phenomenon severely limits the accuracy of synchronization.

We address this issue by defining the output signal *g* as the return-to-zero (RZ) line codes. The output signal *g*(*t*) takes values 1, 0, −1, 0, 1, 0, −1, and 0 during the frames whose frame number mod 8 is equal to 0, 1, 2, and 3, 4, 5, 6, and 7, respectively. It should be noted that the *Off* reference frames are more than previous. However, in our Manchester Encoding scheme, the *Off* frames can compensate for each other in the feedback algorithm. The correlation value $\bar{f(t)g(t)}$ can be sustained by injecting it into a robust recursive low pass filter, whose coefficients should be carefully selected.

**Figure 3.**
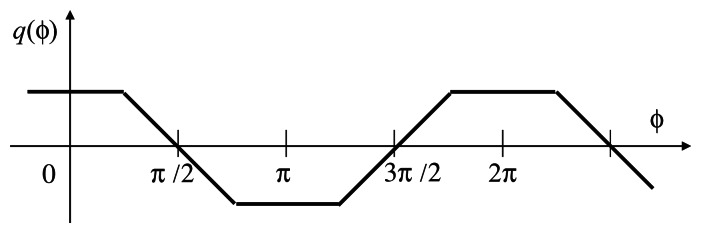
The relations between time correlations and phase difference.

The time correlation value *q*(*ø*) can be seen as a function of the relative phase difference *ø* shown in Figure 3 for the case with full exposure time. Here, the relative phase difference is defined to be zero when the midst time of the reference *On* period and the midst time of the integration period in the frame where *g*(*t*) = 1 coincide. By using the correlation *q* for feedback, the system can converge to the unique stable equilibrium point *ø* = *π*/2. By computing *q* in every frame, negative feedback control is done in real time.

## 3. Frame Index with Temporally Coded Illumination

### 3.1. Motivation of Temporal Encoding

Temporal index in general involves two concepts: one is to add a symbol to the continuous output frames to distinguish a starting time for each image with regard to the network protocol; the other is the concept of pseudo random binary sequence, such as m-sequence, to identify where the image sequences are. The former must modulate a starting time symbol to the regular reference sequence to mark a correspondence time among multiple output images. This paper belongs to the former case. Even if some bright reference unit durations are made dark intentionally, owing to the robustness offered by signal normalization in our previous work, synchronization still works well. Many intelligent encoding strategies can be alternatives to realize the frame index as mentioned before.

In the most ideal situation, one header for the reference signal is enough to distinguish all the output frame indexes, and it is unnecessary to encode the index into a number. However vision sensors do not always work in the ideal situation, because a surveillance system may work all day long, which contains a large number of camera work periods. In the real world situation, it is desirable to index different headers to be different numbers when a large amount of images are taken during different work periods.

### 3.2. Manchester Temporal Encoding Scheme

**Figure 4.**
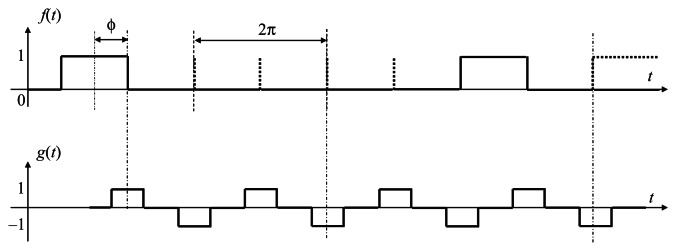
Output signal *g*(*t*), and reference signal *f*(*t*) with selectively darkened reference frames.

Selectively darkening some of the bright reference unit durations may not result in the breakdown of synchronization, owing to the signal normalization algorithm because such missing frames can be regarded as a kind of amplitude fluctuation, if only two bright reference unit durations are made dark within every four reference periods, as illustrated in Figure 4, where a period of the modulated illumination consists of two unit durations in each of which the illumination can be either *On* or *Off* in all of our intensity modulated illumination strategies. Apparently, the feedback amount is still proportional to the sum of time correlation computed based on the bright reference unit durations within the effective correlation window. Therefore, even if the absence of some bright periods can reduce the feedback amount, it only has the same effect as if the reference amplitude was reduced. Theoretically, such decrease of the feedback amount can be compensated by signal normalization.

**Figure 5.**
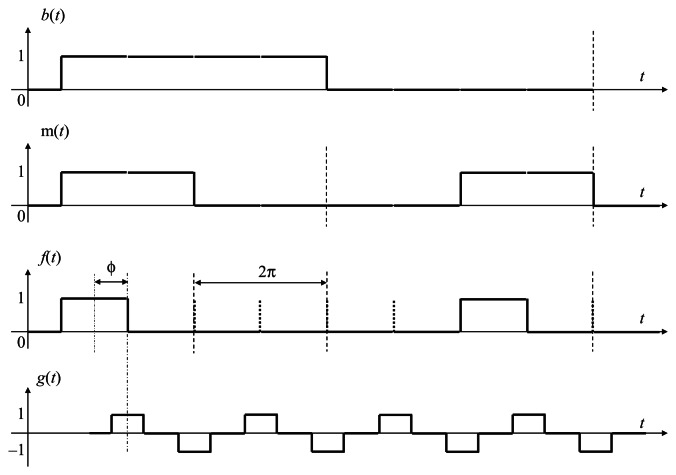
Output signal *g*(*t*), reference signal *f*(*t*), Manchester Encoding sequence *m*(*t*), and binary sequence *b*(*t*).

**Figure 6.**
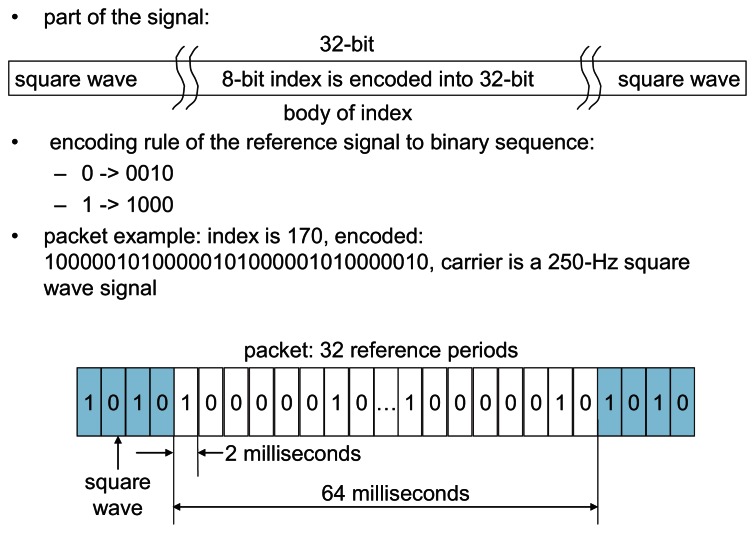
Segment format of Manchester Encoding protocol with 250 Hz square wave carrier.

To distinguish the starting point of the synchronized vision frames, a possible solution is to use a binary encoding strategy by sending a group of binary datagrams, to indicate the starting time of the synchronized images, such as the Manchester Encoding [34]. In Figure 5 the binary sequence *b*(*t*) is encoded in this way, so that a decimal integer range between 0 and 255 are expressed in the binary sequence *b*(*t*) by encoding an 8 encoding units datagram in the reference illumination signal as shown in Figure 6. The index packet includes 16 reference periods, equal to 32 reference unit durations, which takes 64 ms according to the instruction cycle of a vision sensor that is used in the later experiment section.

The output signal *g*(*t*), in fact the vision frame, is modulated as return-to-zero (RZ) line codes, according to signal normalization algorithm. The binary sequence *b*(*t*) expressing the index number is encoded by Manchester Encoding sequence *m*(*t*) for example, (0,1) in *m*(*t*) is decoded as 0 in *b*(*t*), and (1,0) in *m*(*t*) is decoded as 1 in *b*(*t*). A 0 is expressed by a low-to-high transition, and a 1 by a high-to-low transition, according to G.E. Thomas' convention—in the IEEE 802.3 convention. Subordinately, (*off,off*) in *f*(*t*) is decoded as 0 in *m*(*t*), and (*on,off*) in *f*(*t*) is decoded as 1 in *m*(*t*). Therefore, (*on,off,off,off*) in *f*(*t*) is decoded as 1 in *b*(*t*), while (*off,off,on,off*) in *f*(*t*) is decoded as 0 in *b*(*t*). Apparently, the minimum coding unit in *b*(*t*), either 0 or 1, must contain bright reference frame in the temporally encoded illumination. A decimal integer range between 0 and 255 can be expressed in the binary sequence *b*(*t*) by 8 encoding units. The transition property of Manchester Encoding makes this intelligent encoding the most convenient way for the illumination-based synchronization of vision sensors.

### 3.3. Manchester Encoding Feedback Algorithm

In our Manchester Encoding strategy, 4 vision frames duration time is equal to one reference period duration time, as shown in Figure 5 and [35], while the per-frame feedback algorithm still employs quadrature detection in signal normalization, as well as a robust recursive low-pass filter to maintain time correlation *q*_1_(*ø*) taken by the imager.

For each output frame, *i* is the frame number index and *F*(*i*) is the sum of the pixel values obtained within the frame i, as explained in the synchronization algorithm section. After an image is acquired and *F*(*i*) is calculated, *F*(*i*) is stored in one of these 4 variables, *E*_1_, *E*_2_, *H*_1_, and *H*_2_. *E*_1_ and *H*_1_ store pixel values of the output signal *g*(*t*), while *E*_2_ and *H*_2_ store pixel values of the quadrature counterpart of *g*(*t*), which is not demonstrated apparently this time as in the signal normalization algorithm. The values are updated according to the following principle:

(2) $$E_{1}=F[i],\;\text{when}\;(i\;\text{mod}\;8\;)\;=\;0,\;4$$

(3) $$E_{2}=F[i],\;\text{when}\;(i\;\text{mod}\;8\;)\;=\;1,\;5$$

(4) $$H_{1}=F[i],\;\text{when}\;(i\;\text{mod}\;8)\;=\;2,\;6$$

(5) $$H_{2}=F[i],\;\text{when}\;(i\;\text{mod}\;8)\;=\;3,\;7$$

Using these variables, in each frame, the discrete-time low-pass filters giving time correlation *q* [*i*] at frame *i* are implemented as first-order recursive filters with an eight-frame moving window

(6) $$q_{1}[i]=a_{1}\cdot q_{1}[i-1]+a_{2}\cdot (E_{1}-H_{1})$$

(7) $$q_{2}[i]=a_{1}\cdot q_{2}[i-1]+a_{2}\cdot (E_{2}-H_{2})$$

where *q*_1_ [*i*] and the correlation of its quadrature counterpart, *q*_2_ [*i*], are achieved at the end of each frame. The length of the non-integration period *τ_nonint_* is negative-feedback controlled every frame in accordance with the time correlation *q*_1_ [*i*] and the *normalizer* max (|*q*_1_[*i*]|, |*q*_2_[*i*]|), while the length of the integration period is fixed. This negative feedback control is the same to our previous research [33, 35]. The coefficients of the IIR LPF *a*_1_ and *a*_2_ are set to 0.1 and 0.9 respectively and arbitrarily, by analyzing the convergence time and the jitters, as well as the undershoot and overshoot of system before convergence.

**Figure 7.**
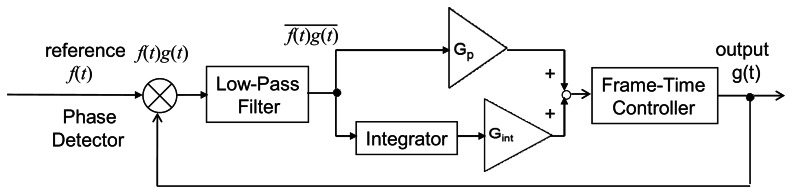
Block Diagram of PLL with PI controller.

To remove the steady-state residual phase error caused by frequency mismatch, a PI (proportional-integral) controller is added to PLL as shown in Figure 7. An integral term is added to Equation (8) as

(8) $$\tau_{\text{nonint}}[i]=\tau_{0}+\frac{G_{p}\cdot q_{1}[i]}{\max(|q_{1}[i]|,|q_{2}[i]|)}+G_{i}\sum_{j=0}^{\infty }\frac{q_{1}[i-j]}{\max(|q_{1}[i-j]|,|q_{2}[i-j]|)}$$

where *τ*_0_ is a constant set to 0.2 ms, and *G*_p_ and *G*_i_ are constant values optimized by simulation. The adjustment resolution of *τ*_nonint_ is 100 ns, which is the instruction cycle of the proposed vision sensors.

## 4. Simulation

The simulation is carried out to explore feasible system coefficients. A high-speed vision sensor is modeled to operate at 1000 Hz frame rate and with 64 × 64 pixels, which requires 250 Hz modulated illumination like [35] and thus decides the central frequency of the Manchester Encoding scheme, because if *f*(*t*) is 250 Hz, correspondingly *b*(*t*) is 62.5 Hz. The frame rate and the number of pixels are decided with respect to those of the high-speed vision sensors used in real world experiments.

**Figure 8.**
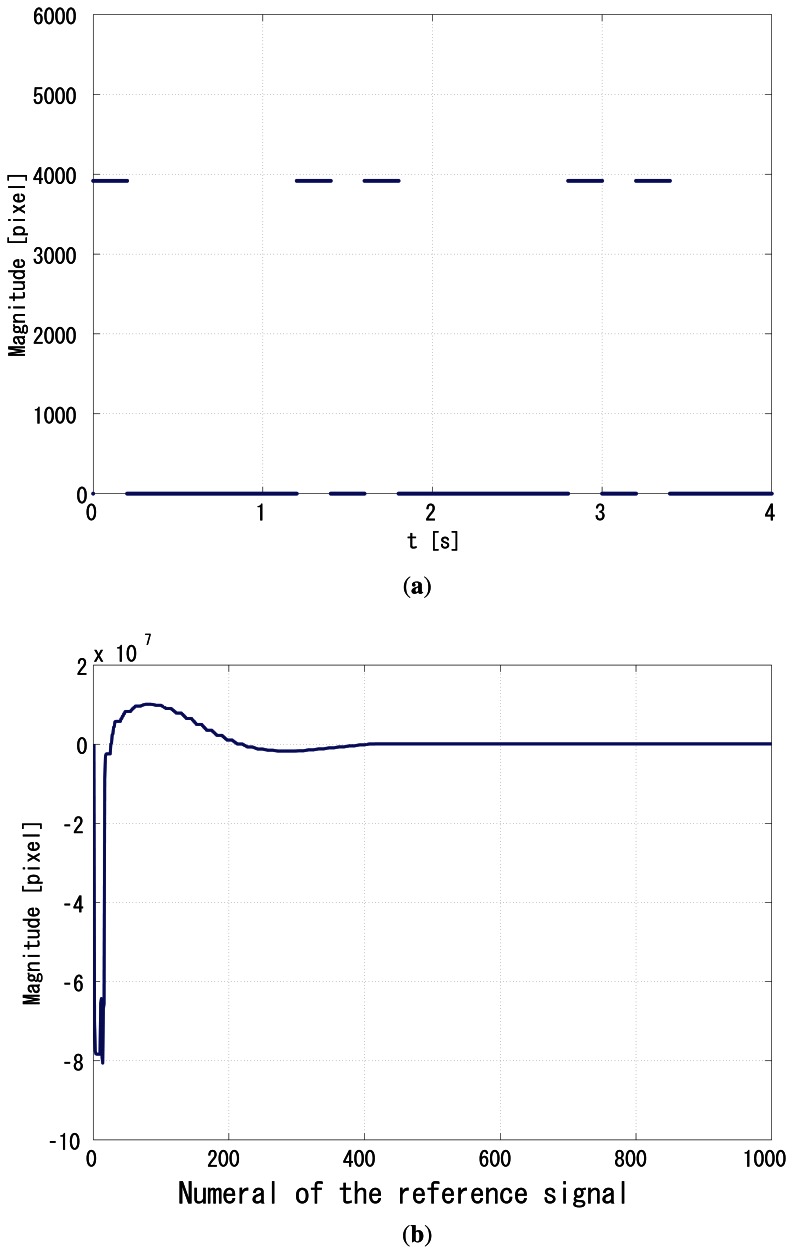
Simulation results, (**a**) *f*(*t*), (**b**) *q̅*_1_(*t*).

The coefficients *a*_1_ and *a*_2_ are set to 0.9 and 0.1 intentionally, to make the LPF robust enough to sustain the previous correlation values. The unit of gain is s/pixel, because *q* [*i*] is in the dimension of the pixel value multiplied by the number of pixels, and the pixels value is dimensionless. Figure 8(a) shows the reference signal modulated in the Manchester Encoding scheme, which is a specifically modulated sequence (1,0,0,0,0,0,1,0) and repeats itself every 32-reference period. It expresses a 16-period self-repeating Manchester Encoding (1,0,0,1), which expresses a 8-period (1,0) binary sequence.

**Figure 9.**
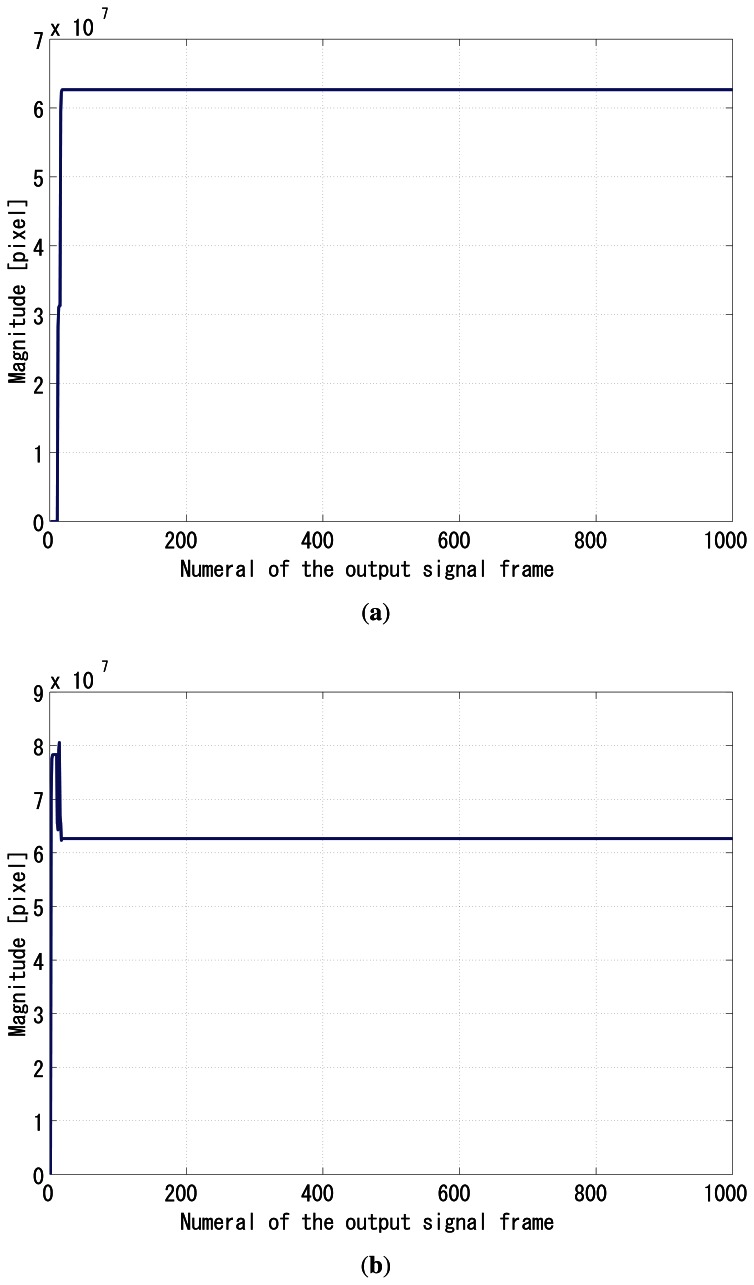
Simulation results, (**a**) *q̅*_2_(*t*), (**b**) normalizer.

**Figure 10.**
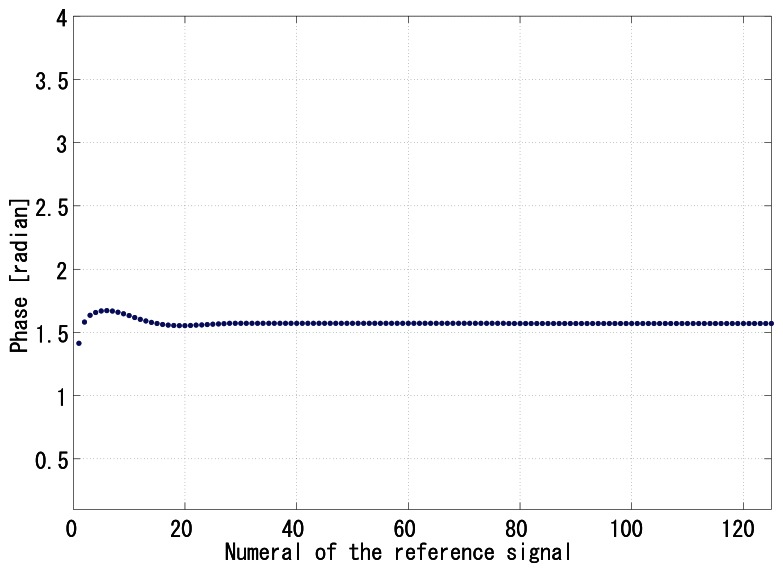
Phase shift between *f*(*t*) and *g*_1_(*t*).

The gain *G_p_* was set to 64, and *G_i_* was set to 1. Figure 8(b) shows the time correlation value of the output signal *g*_1_(*t*), and Figure 9(a) shows the correlation of *g*_2_(*t*). Figure 9(b) shows the normalizer. Figure 10 shows the relative phase between *f*(*t*) and *g*_1_(*t*). It can be seen that the system immediately converged to the *π*/2 relative phase and became stable thereafter. Apparently, the PI feedback helps to reduce the discrepancy between *π*/2 and the real phase, which corresponds to the steady-state error.

This time, it is unnecessary to evaluate system performance again, because the temporal modulation strategy can be directly transplanted to the existing signal normalization algorithm introduced in our previous work, by intentionally adjusting the coefficients of LPFs to increase the feedback effect of previous frames so as to maintain synchronization. This assumption has been tested and proved correct in the synchronization algorithm section in this paper.

## 5. System Implementation

**Figure 11.**
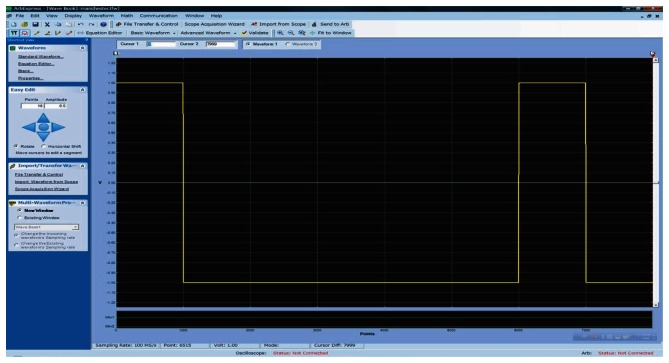
Waveform Creation Software for Tektronix Signal Generators.

A temporally modulated Manchester Encoding sequence carries sequential information in every index segment. Due to the irregular waveforms of the reference signal, this time we modulate the LED reference illumination in advance and store the waveform coding within Tectronics AFG3102 with the help of MATLAB. Specific wave data according to different indexes and monitoring lengths can be generated in the format of CSV files by MATLAB beforehand and is then converted to TFW files for the arbitrary waveform generator. The snapshot of a sequence of self-repeating two reference periods (1,0,0,0,0,0,1,0) generated by wave generator is shown in Figure 11.

### 5.1. Temporal Modulation of LED Reference

First of all, the most interesting case to test is the Manchester code itself, especially for the high-speed vision sensor operating at 1,000 Hz, where a Manchester indexed header can pass by in a flash prior to the ongoing regular square waveform. Therefore, the feasibility of Manchester Encoding strategy should be tested independently and completely. A meaningful experimental length should totally be made up by the Manchester Indexed header. If this strategy succeeds, an indexed header with regular square wave can also be proved feasible, according to our previous research. Corresponding to the later first experiment, the reference illumination is solely made up by 16 reference period long self-repeating periods in *f*(*t*). The typical Manchester Encoded reference signal (1,0,0,0,0,0,1,0) in our scheme, where *G_p_* is set to 64 and *G_i_* is set to 32, coincides with the simulation results. Robust coefficients of the IIR LPF in the feedback algorithm are set to 0.9 and 0.1, respectively. The experimental result will be generalized in the first line in Table 1.

Consequently, another extreme case, corresponding to the second experiment in later section, should also be tested to prove feasibility of the transition virtue of Manchester Encoding for our research. An 8-period (0,0,0,0,0,0,0,0) symbol standing for decimal 0 can be encoded to be 32-reference periods long (0,0,1,0) self-repeating frames in *f*(*t*). If such fewest bright reference unit durations sequence can synchronize successfully, the index range from [0, 255] will be proved. This experimental result will be generalized in the sixth line in Table 1.

### 5.2. Illumination Brightness

It is desirable to know whether the Manchester Encoding synchronization is totally robust to background light, such as fluorescent lamp and reflected light. Two other groups of tests are carried out at night laboratory environment, leaving only the effect of fluorescent lamp, which will be shown in the second and the third lines in Table 1. The modulated LED illumination is reflected onto the lens of the vision sensor by a white reflector. In the first group, the illumination of fluorescent lamp is strong, about 402 lx. In the second group, the illumination of fluorescent lamp is weak, about 156 lx.

### 5.3. Highly Dynamic Scenes

Finally, to what extent the synchronization is tolerable to the dynamic fluctuation of brightness is also investigated, with a high-speed rotating target in the scene of vision sensor, inspired by the real-time measurement with the vision sensor [36,37]. One of the fundamental applications of the high-speed vision chip is to measure high-speed rotation in the industry field. Using the high-speed vision chip, advantages of real-time visual measurements become evident. Targets moving at high speed and with irregular motion can be measured with high precision. Therefore, it is of primary importance to evaluate the synchronization performance with high-speed targets of dynamic fluctuation of brightness by mimicking the scene in those measurements.

**Figure 12.**
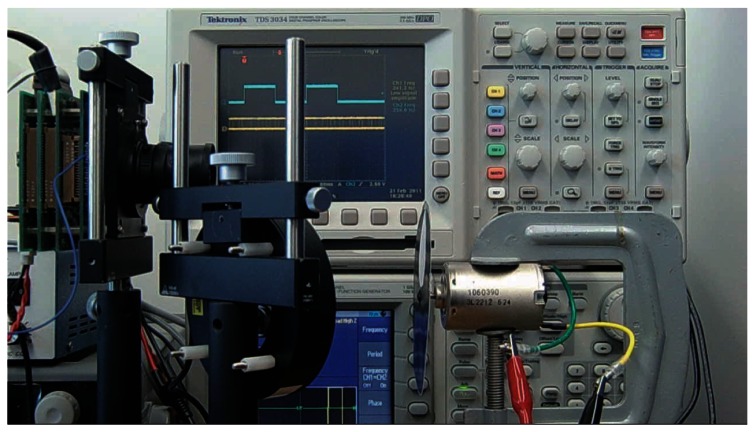
Experiment scene with intensity modulated illumination reflected from the high-speed rotating white marked target.

The high-speed rotation is driven by a DC motor, MABUCHI MOTOR RS-540SH. When the input power reaches the peak value 7.2 V, the rotation speed can be as high as 14,500 rpm, equal to 242 Hz, which is high-speed enough to test the robustness of the signal normalization technique. The true rotation speed is obtained by another high-speed camera serving as a decoder, by measuring the rotation, to find out the real linear relation between the input voltage to DC motor and the rotation speed. The experimental scene is shown in Figure 12.

**Figure 13.**
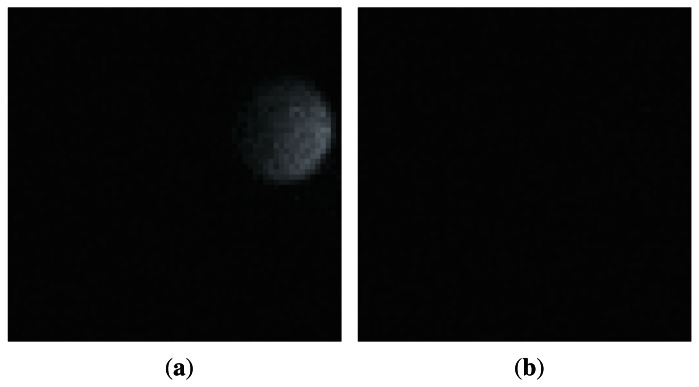
Images of the rotating target taken by vision chip, (**a**) white circle inside visual field; (**b**) white circle outside visual field.

The diameter of the black circle target is 10 cm, with a small white circle, whose diameter is 4 cm, centered along the radius of the black target. If the target is located in front of the vision chip with high-speed rotation, the white circle will appear inside or outside the vision field, and thus generate the fluctuant reference amplitude because the modulated illumination is reflected into the lens of vision chip by the target. The 64 × 64 pixels images taken by vision chips can genuinely reflect such amplitude fluctuation. In Figure 13(a), the maximum pixel intensity is 37, the average pixel intensity is 2.4, and the sum of pixel intensity is 9960. In Figure 13(b), the maximum pixel intensity is 7, the average pixel intensity is around 0.81, and the summation of pixel intensity is 3,338. The experimental result will be generalized in the fourth line in Table 1.

## 6. Experiments

Several experiments were carried out on the vision chip system [1] to evaluate the system performance, which are generalized in Table 1. The last two columns show the synchronization results, such as state and jitters, under different conditions.

### 6.1. Index Range and Detection

**Figure 14.**
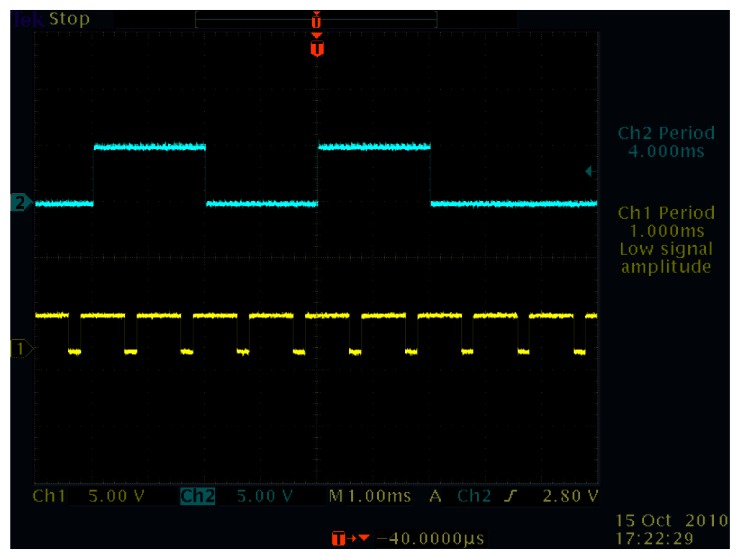
Peak-to-peak jitters of the output signal to index 170, 24 *μ*s.

**Figure 15.**
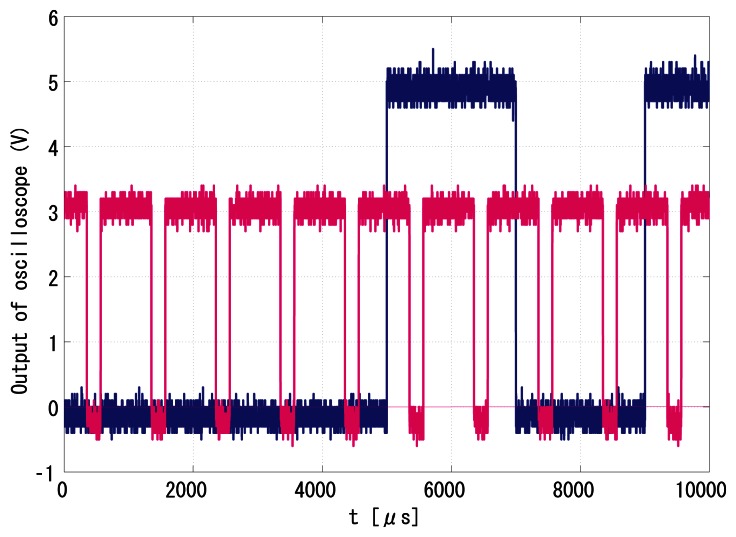
Successful synchronization result of the index 170.

With regard to the introduction in Section 5.1, the Manchester Encoded indexes were tested for feasibility. Figure 14 and Figure 15 show the successfully synchronized results of the index 170. The blue signal is the reference illumination signal recorded and exported from the oscilloscope and is plotted out by MATLAB. The red signal is the output vision frame signal exported from the oscilloscope in the same experiment and is plotted by MATLAB.

**Figure 16.**
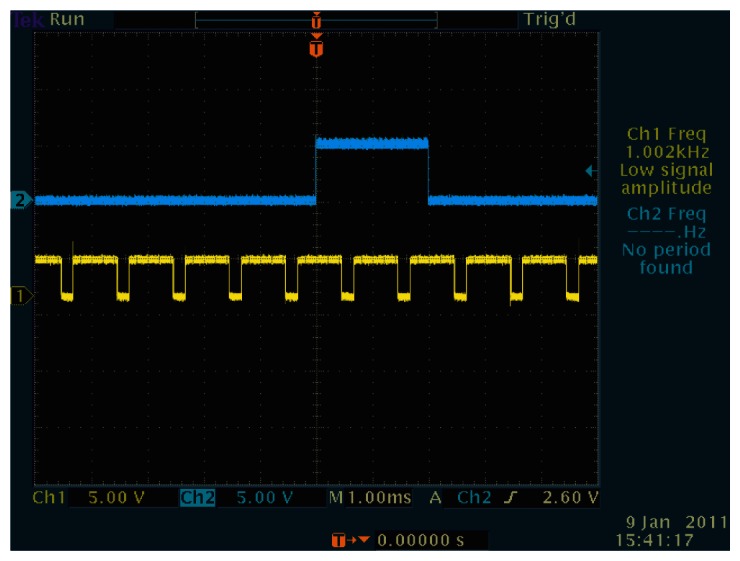
Peak-to-peak jitters of the output signal to index 0, 24 *μ*s.

**Figure 17.**
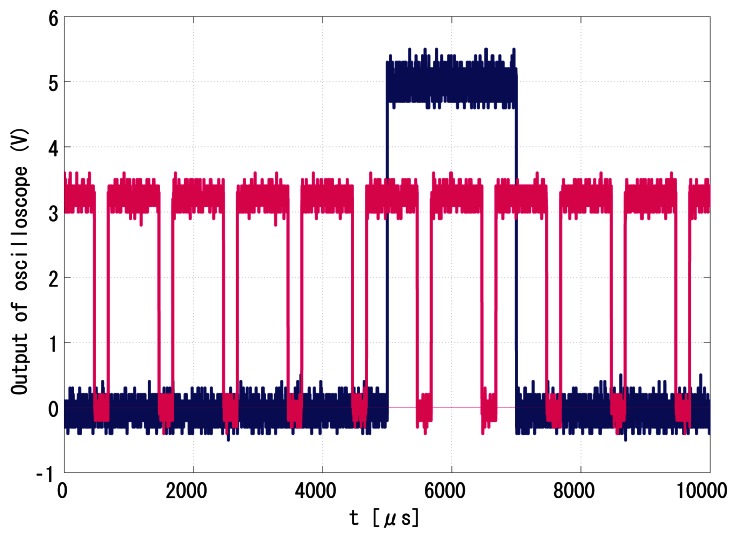
Successful synchronization result of the index 0.

Figure 16 and Figure 17 show the successfully synchronized results of the index 0. Both output signals were successfully synchronized to the reference signal with *π*/2 relative phase shift and twice the frequency. The peak-to-peak jitters of the output signal measured by the oscilloscope were around 24 *μ*s, which are only around 1.2% of the reference period and thus 0.12-rad phase error at worst, which is satisfactory enough for practical use.

We used to carry out MATLAB simulations to evaluate system performance by importing a group of images taken by a USB WebCam. The average pixel value of each image was calculated simultaneously by OpenCV when it was acquired. Similarly, in this paper, the average pixel value of each image taken by the vision chip can be calculated offline by image processing functions in MATLAB. Even if there is background light as shown in Figure 12, the effects of bright or dark unit durations as incident light are apparently different in forms of the sum/average pixel values of the output images. The average pixel values of images taken in the bright durations are higher than those in the dark unit durations.

Firstly, the starting position of an index packet can be distinguished from the regular square wave carrier by comparing the average pixel values of images. Previously, the reference signal is intensity modulated in standard square wave form, so in the locked state the average pixel value of each image is the same. Now in the Manchester Encoding strategy, some bright unit illumination durations are darkened on purpose to carry index information. In the ideal locked state shown in Figure 5, when the phase difference between the illumination and the frame signal is exactly *π*/2, every other frame lies in the middle of single unit illumination duration, either bright or dark with regard to the encoding law of specific index. The header is carried by a group of intensity modulated illumination in standard square wave. Once a sequence of output image with low pixel values emerges compared with regular average pixel values, the starting position of an index is confirmed.

Secondly, although the output image cannot directly give the index information, the binary numerical value carried by the Manchester modulated illumination can be obtained from the segmented sequence indirectly. After the average pixel value of each output image of the index segment is calculated in order, the image with regular pixel value, when the midst time of its integration period coincides with the midst time of a period of the modulated illumination, is decoded as 1. The image with comparable low pixel value, when the midst time of its integration period coincides with the midst time of a period of the modulated illumination, is decoded as 0. The binary numerical value of the output frame in *g*(*t*) is the same to the index information carried by the Manchester encoded illumination in *f*(*t*). This is how to recover the index from the temporal information presented in the image sequence. Please notice that the comparison of average pixel values is a relative process for each output sequence, because different background light and experimental conditions can affect the pixel values globally. Furthermore, for high-speed vision chips running at 1,000 Hz, background light, as well as other sudden flash of light, does not affect index recovery.

### 6.2. Effective Intensity of Reference Illumination

**Figure 18.**
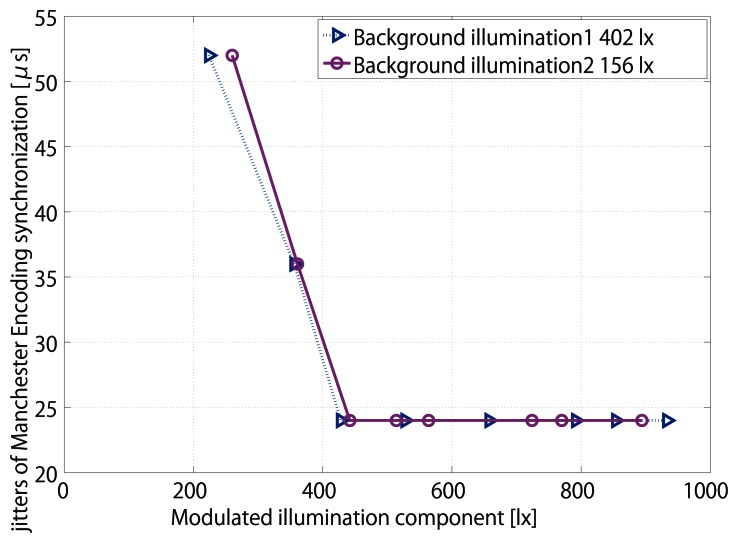
Jitters of different modulated ratio in total illumination of Manchester Encoding synchronization.

The LED intensity is measured by a luminometer, with the minimum jitter being 24 *μ*s. In the first test, when the modulated illumination is 9 lx within the total 411 lx illumination, 2.2%, synchronization is lost. Jitter is 24 *μ*s at minimum and can increase up to 52 *μ*s. In the second test, when the modulated illumination is 18 lx within the total 174 lx illumination, 10.3%, the synchronization is lost. Jitter is 24 *μ*s at minimum and can increase up to 52 *μ*s. The minimum jitters are the same according to different background lights in Figure 18.

### 6.3. Robustness in Highly Dynamic Scenes

**Figure 19.**
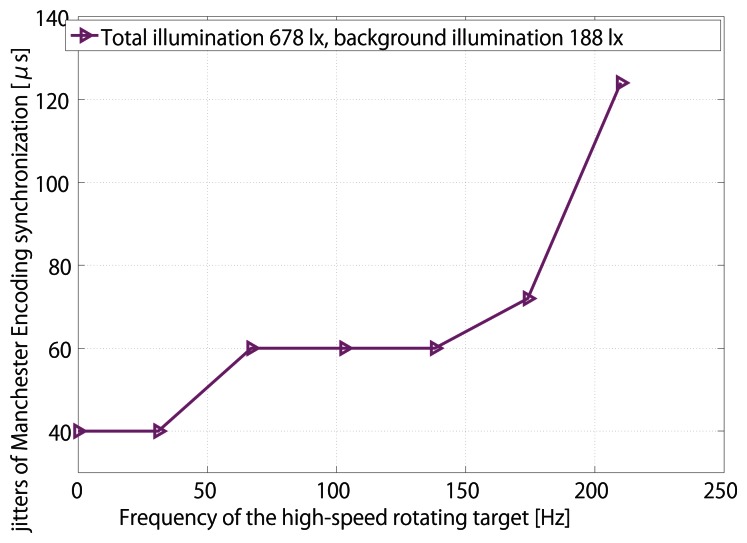
Synchronization Jitters of different rotational speeds of target.

The relation between the rotation speed of target and the synchronization jitters is shown in Figure 19. Owing to the virtue of signal normalization, jitters keep steady only after the rotation speed of the target reaches 100 Hz. However, this time, with the help of robust LPF coefficients, jitters indeed reach a platform, 60 *μ*s, after the rotation speed of the target reaches 138 Hz. Finally, when the rotation speed exceeds 228 Hz, almost the same as the modulated reference frequency, synchronization is totally lost. Therefore, we come to the conclusion that the robustness of the Manchester Encoding scheme is also satisfactory.

## 7. Conclusions

By encoding the reference illumination in the Manchester way, the successfully synchronized video sequences can be properly indexed. However, there are two intrinsic weaknesses in this method along with the all advantages mentioned above. Firstly, the visibility of scene has been affected by the new Manchester Encoding scheme. In our previous algorithms, the visibility was not or just slightly influenced because half period of every frame is almost always illuminated in the locked state. Comparably, according to this temporally encoding mechanism, due to the disappearance of some bright reference unit durations, in the locked state, some vision frames of the index symbol will be completely not illuminated. Fortunately, such drawback can be compensated by employing background light, therefore allowing the index to be distinguished by comparing the average brightness of images. Secondly, the existence of the index symbol by irregularly temporally modulating the reference signal can engender slightly larger jitters. However this temporary phenomenon is neither permanent nor fatal, only occurring simultaneously with the index but not within the whole work period of vision sensors in both ideal and practical situations.

## Figures and Tables

**Table 1.** Experimental conditions and results.

	**Light source**	*G_p_*	**LED**	**Distance**	**Index**	**Jitters**	**State**
1	direct illumination	64	743 1x	0.5 m	170	24	Locked
2	indirect illumination	64	402 1x	0.3 m	170	24	Locked
3	indirect illumination	64	156 1x	0.3 m	170	24	Locked
4	indirect illumination reflected from high-speed rotating target	64	678 1x	0.3 m	170	40	Locked
5	direct illumination	64	1,099 1x	0.5 m	170 with square-wave carrier	30	Locked
6	direct illumination	64	725 1x	0.5 m	0	24	Locked
